# Selective Oxidation of Alcohols through Fe_3_O_4_@SiO_2_/K_2_CO_3_‐Glycerin Deep Eutectic Solvent as a Heterogeneous Catalytic System

**DOI:** 10.1002/open.202200172

**Published:** 2022-12-01

**Authors:** Sepideh Abbasi, Mohammad Reza Naimi‐Jamal, Shahrzad Javanshir, Akbar Heydari

**Affiliations:** ^1^ Research Laboratory of Green Organic Synthesis & Polymers Department of Chemistry Iran University of Science and Technology (IUST) 16846-13114 Tehran Iran; ^2^ Heterocyclic Chemistry Research Laboratory Chemistry Department Iran University of Science and Technology 16846-13114 Tehran Iran; ^3^ Chemistry Department Tarbiat Modares University 14155-4838 Tehran Iran

**Keywords:** alcohol oxidation, deep eutectic solvent, green chemistry, heterogeneous catalyst, magnetic nanoparticles

## Abstract

K_2_CO_3_/Glycerin as a deep eutectic solvent (DES) was anchored covalently onto functionalized magnetic nanoparticles and showed a significant activity towards the oxidation of various alcohols under mild conditions with a short reaction time and good to high yield. A combination of the magnetic nanoparticles and deep eutectic solvent offers a novel, green, reusable catalyst with easy separation. Also, the catalyst structure was well characterized using techniques such as FT‐IR spectroscopy, XRD, SEM, TGA, BET, VSM, TEM, and energy‐dispersive X‐ray spectroscopy (EDS).

## Graphical abstract

**K_2_CO_3_/Glycerine as a deep eutectic solvent** was anchored covalently onto magnetic Fe_3_O_4_@SiO_2_ core‐shell nanoparticles. This heterogeneous catalyst was effectively used for the selective oxidation of alcohols to corresponding carbonyl products with up to 95 % yields.

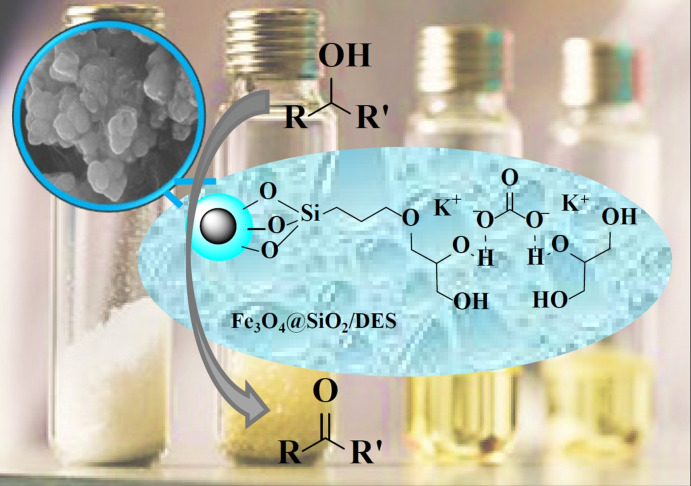

## Introduction

Oxidation reactions, especially selective oxidation of alcohols using heterogeneous catalysts, have received growing attention and offer a green and sustainable route to value‐added chemicals.^[1]^ In the last few years, much work has been devoted to metal‐catalyzed oxidation reactions due to unique features such as high turnover frequency and selectivity, but still involved with problems such as the difficulty of purification, stability in the reaction conditions, and complicated workup processes.^[2]^ On this account, tremendous efforts are devoted to obtaining alternative compounds and structures with similar properties to metal‐containing catalysts. In this regard, metal‐free catalytic systems have been highly considered in chemical research owing to their extraordinary capabilities such as (i) ease of preparation, (ii) low cost, (iii) stability, and (iv) high durability.^[3]^ So, it is worth having a great deal of focus on these systems.

Several different protocols and efficient methods have been suggested for oxidation reactions, especially for the selective oxidation of primary and secondary alcohols to corresponding products using a few‐layered phosphorene–graphitic carbon nitride nano heterostructure SO_2_F_2_/K_2_CO_3_ in DMSO, CF_3_SO_2_Na/LED, P‐doped carbon, hexachloroacetone, etc.^[4]^ The majority of the above‐stated methods suffer from many disadvantages, including the use of toxic reagents, volatile solvents, or expensive catalysts.

Perfluorinated solvents, among the used solvents for oxidative reactions, are essential due to their high oxygen solubility,^[5]^ on the other hand, the use of media that plays a catalytic role, in addition to being in line with the principles of green chemistry, could be another unique solution. Recently, the use of ionic liquids, due to their extraordinary ability to form hydrogen bonds, has received much attention for a variety of oxidation reactions, especially the C−H oxidation.^[6]^ Deep Eutectic Solvents (DESs), as new emerging analogs of the ionic liquid have attracted increasing attention in a variety of chemical reactions due to advantages such as convenient preparation, low cost of raw materials, biocompatibility, low toxicity, and no sensitivity towards moisture.^[7]^ Generally, DESs are formed by combining two salt components such as quaternary ammonium and a hydrogen bond donor in several specific ratios.^[8]^ The most common DES is composed of choline chloride (ChCl) and urea with a 1 : 2 molar ratio.^[9]^ Recently, glycerol and potassium carbonate has been used in different molar ratios to obtain a new glycerol‐based DES system.^[10]^ DESs have been used in many organic syntheses such as acetylation of carbohydrates and cellulose, selective N‐alkylation, phthalimide synthesis, etc.^[11]^

According to the literature, DESs are used as a dual catalyst/solvent in organic synthesis, so they could be considered homogeneous catalysts.[^8b^, ^12^] To attain high catalytic activity, heterogenization of homogeneous catalysts could be a common way. Besides other solid supports, MNPs with good stability, easy functionalization, surface properties, and easy separation by an external magnet have emerged as attractive support for the immobilization of homogeneous functional groups and metal nanoparticles in organic catalytic transformations.^[13]^ Immobilization of DESs forming from inexpensive, widely available, and environmentally‐friendly starting materials on magnetic nanoparticles could provide green, efficient, biodegradable, and biocompatible heterogeneous catalysts. The oxidation of alcohols to the corresponding aldehydes and ketones is considered one of the most important transformations in organic synthesis,^[14]^ Therefore, using of these new catalysts could utilize both in the laboratory‐scale and industrial processes for these reactions.

Herein, we describe a highly economical method for the fabrication of active hybrid magnetic nanocomposites modified with K_2_CO_3_/Gly DES and investigate their catalytic activity for the selective oxidation of alcohols to their corresponding carbonyl compounds under mild conditions.

## Results and Discussion

In the present work, Fe_3_O_4_ nanoparticles were synthesized in a chemical co‐precipitation method and subsequently coated with tetraethyl orthosilicate (TEOS) to obtain the Fe_3_O_4_@SiO_2_. Then, the Fe_3_O_4_@SiO_2_ was functionalized by as‐synthesized 3‐iodopropyltrimethoxysilane **(**IPS).

**Scheme 1:**
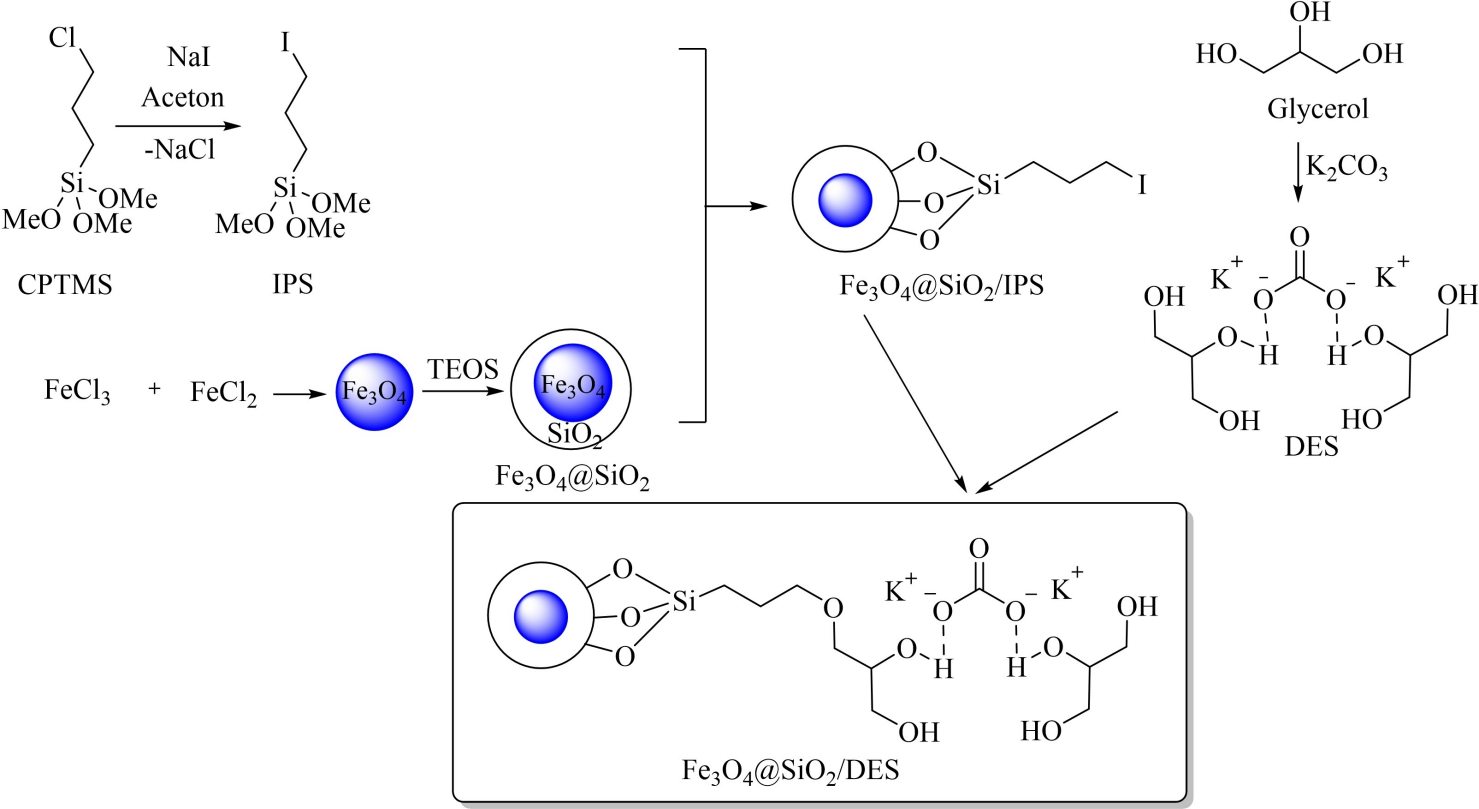
The schematic synthetic procedure for Fe_3_O_4_@SiO_2_/DES.

K_2_CO_3_‐Glycerol DES has been engrafted on the Fe_3_O_4_@SiO_2_/IPS nanoparticles as illustrated in Scheme 1.

**Figure 1:**
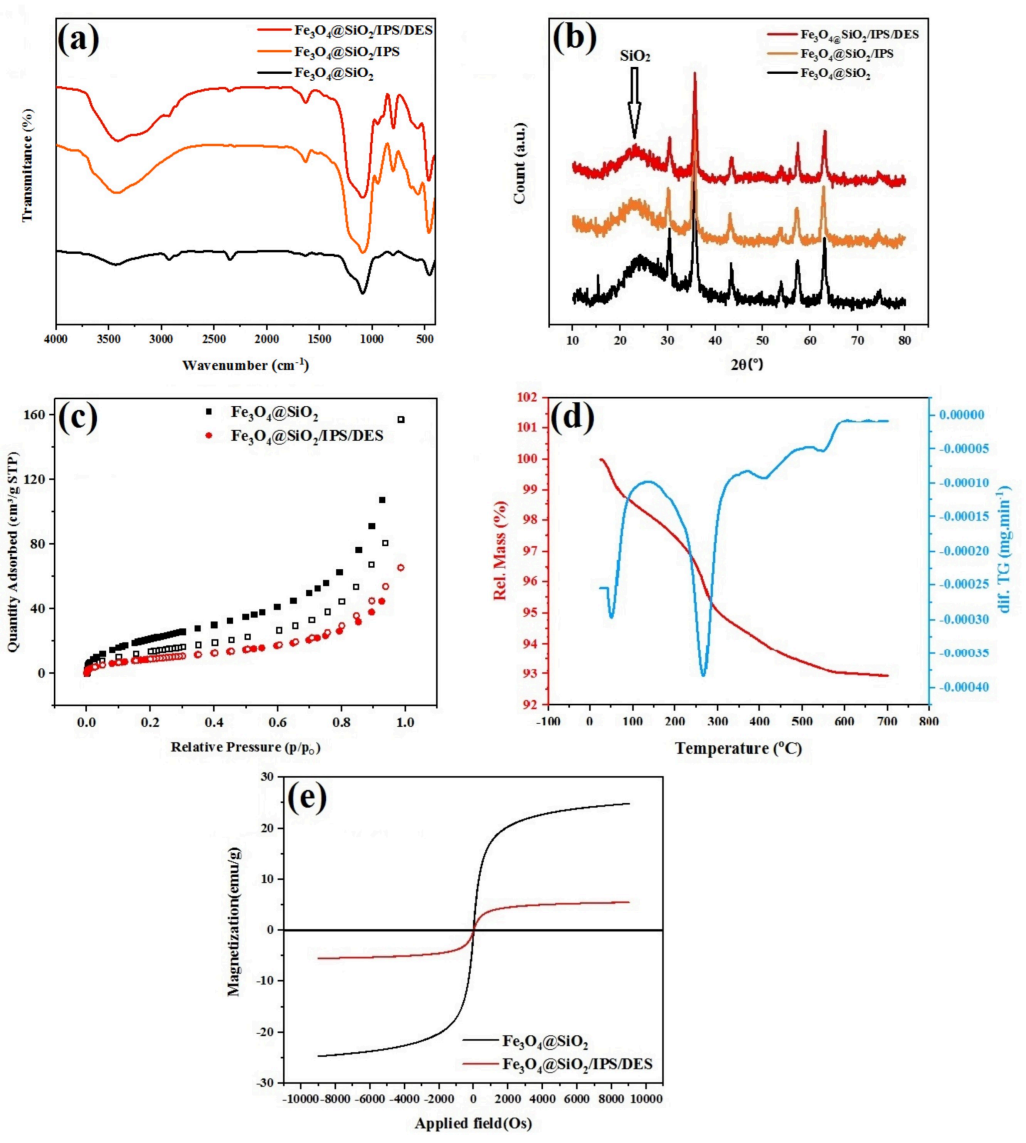
(a) FTIR of tandem steps of catalyst preparation. Fe_3_O_4_@SiO_2_, Fe_3_O_4_@SiO_2_/IPS, and Fe_3_O_4_@SiO_2_/DES. (b) XRD patterns of three major steps for preparation of immobilized DES onto linker‐aided nanoparticles. (c) N_2_ adsorption‐desorption isotherms Fe_3_O_4_@SiO_2_ and Fe_3_O_4_@SiO_2_/DES. (d) TGA and dif.TG of Fe_3_O_4_@SiO_2_/DES. (e) Vibrating sample magnetometer (VSM) of Fe_3_O_4_@SiO_2_ and Fe_3_O_4_@SiO_2_/DES.

The catalyst characterization was performed using several techniques: FT‐IR spectroscopy, XRD, SEM, EDX, TGA, BET VSM, and TEM. The FTIR spectra of the Fe_3_O_4_@SiO_2_, Fe_3_O_4_@SiO_2_/IPS, and Fe_3_O_4_@SiO_2_/DES, were shown in Figure 1a.

In the FTIR spectrum of the Fe_3_O_4_@SiO_2_, the major peaks appeared around 568 cm^−1^ and 1085 cm^−1^ are respectively corresponding to Fe−O and Si−O stretching vibrations. FTIR spectra of both Fe_3_O_4_@SiO_2_/IPS (Figure 1a, in orange) and Fe_3_O_4_@SiO_2_/DES (Figure 1a, in red) also show the same peaks for Fe−O and Si−O vibrations. The coating of IPS on the surface of magnetic nanoparticles can be confirmed by the bands at 1040 cm^−1^, and 2925 cm^−1^ assigned to the C−O, and aliphatic C−H stretching vibrations, respectively. The characteristic peaks of the final catalyst are seen at 1405 cm^−1^ and 1657 cm^−1^, which were attributed to the C−O and C=O symmetric and asymmetric vibrations of carbonate group and the peak at 630 cm^−1^ corresponding to the stretching vibrations of K−O. These results indicate that magnetite nanoparticles were successfully coated with the IPS and then the K_2_CO_3_‐Glycerol DES.

The XRD pattern of the crystalline structure of the prepared Fe_3_O_4_@SiO_2_/DES was shown in Figure 1b. The characteristic peaks are compatible with the standard Fe_3_O_4_ (corresponding to the JCPDS 75‐0033 reference), and the crystal structure of pure Fe_3_O_4_ remains unchanged after the immobilization of SiO_2_ and DES. The broad peak appearing at 21.05° is attributed to Si, which is shifted by adding a viscous DES. Similar to a previously reported sugar‐aided catalyst, this shift can be ascribed to the reflection of X‐ray arrays inside such a mucous environment after transmitting across crystalized nanoparticles Fe_3_O_4_@SiO_2._ ^[15]^

The N_2_ adsorption‐desorption isotherms for fresh Fe_3_O_4_@SiO_2_ and Fe_3_O_4_@SiO_2_/DES were also employed (Figure 1c). The adsorption isotherm is of type IV, and the appearance of the hysteresis loop shows mesopores structure in the sample.

The structural stability of Fe_3_O_4_@SiO_2_/DES was also determined using the thermal gravimetric (TGA) technique as well as the differential thermal analysis (DTA). The first stage of weight loss at about 100 °C, was associated with the removal of possible solvents (organic and water) used in catalyst preparation. The second and the third steps of weight loss occurred at about 300 and 400 °C, which is the onset of the structural degradation of the DES and separation of the propyl part of the catalyst, respectively (Figure 1d).

The magnetic properties of the Fe_3_O_4_@SiO_2_ and Fe_3_O_4_@SiO_2_/DES were studied by the vibrating sample magnetometer (VSM) as shown in Figure 1e. Based on Figure 1e, the magnetization saturation (Ms) of the Fe_3_O_4_@SiO_2_/DES was about 5 emu ⋅ g^−1^ which is lower than that of Fe_3_O_4_ NPs with (about 25 emu ⋅ g^−1^) due to immobilization and coating with a high‐loaded DES layer as a non‐magnetic section.

**Figure 2:**
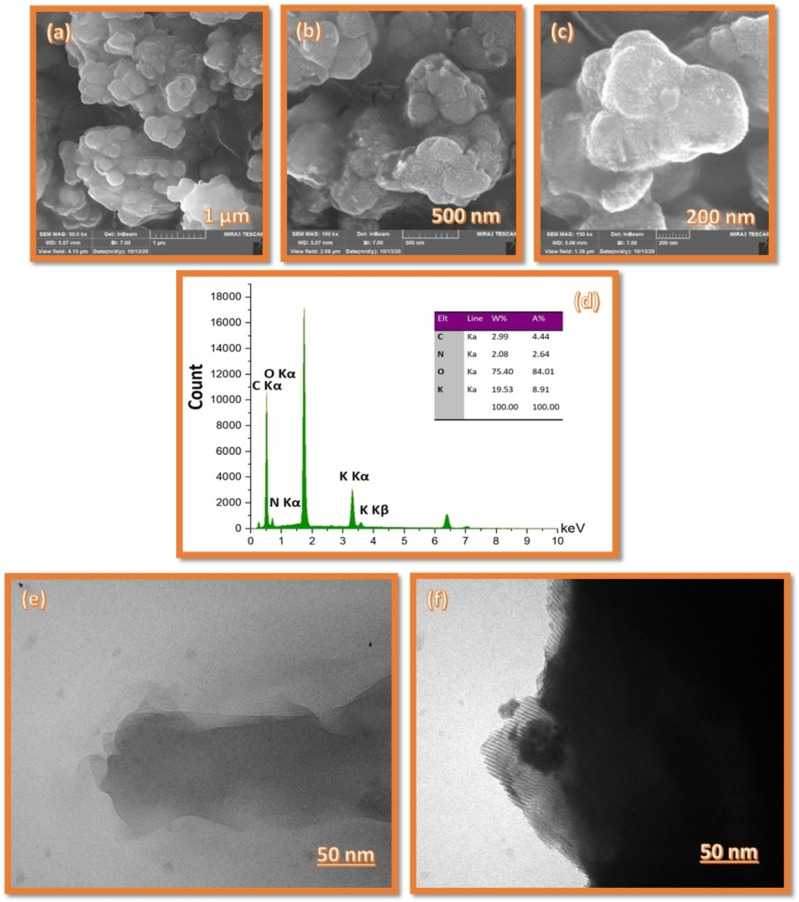
(a–c) FE‐SEM images, (d) Energy Dispersive Analysis of X‐ray Diffraction (EDAX), and (e–f) Transmission electron microscopy (TEM) images of Fe_3_O_4_@SiO_2_/DES nanoparticles.

The particle size and shape of Fe_3_O_4_@SiO_2_/DES nanoparticles were investigated by scanning electron microscopy (SEM) and transmission electron microscopy (TEM). Figures 2a–c illustrate the Field Emission SEM (FE‐SEM) images of the Fe_3_O_4_@SiO_2_/DES nanoparticles. According to these images, the surface morphology of the Fe_3_O_4_@SiO_2_/DES nanoparticles is uniform with a spherical shape. The average size of the nanoparticles was estimated at around 10 to 75 nm with a moderated degree of agglomeration, as shown in the 5 μm‐scaled SEM image. The 200‐nanometer‐scale image shows the nanoparticles being surrounded by a honey‐like mucus. For more surveys and more detailed images of the catalyst, TEM images were taken as exhibited in Figures 2e and 2 f. These images indicate that the prepared catalyst has particles of nearly spherical and rectangular shapes with average co‐agglomerated nanoparticles′ size of around 75 nm.

The energy‐dispersive X‐ray analysis (EDX) spectrum of Fe_3_O_4_@SiO_2_/DES nanoparticles also indicates the presence of potassium (8.9 %) and carbon (4.4 %) as two distinct elements in this DES and confirms grafting of the DES with the modified magnetic nanoparticles. Importantly, it was found that there is no iodine element in the catalyst structure demonstrating that DES anchored covalently onto functionalized magnetic nanoparticles. The low amount of the N fraction can be described as the trapped nitrogen of air inside of first DES as micro‐bubbles (Figure 2b).

To investigate the catalytic performance of the prepared Fe_3_O_4_@SiO_2_/DES, the oxidation reactions were investigated. To obtain an optimum condition for different types of reactants, the amount of the catalyst, temperature, the presence or absence of solvent, and also the type of oxidants have been studied.

Oxidation of benzyl alcohol was carried out as a model reaction in the presence of Fe_3_O_4_@SiO_2_/DES as the catalyst, and hydrogen peroxide as a green oxidant. In an initial experiment, no product was observed when the mixture was heated to 80 °C for a long time in the absence of the catalyst. The reaction was then carried out in the presence of pure Fe_3_O_4_@SiO_2_ nanoparticles and neat DES separately and the desired product was yielded at 28 % and 32 %, respectively. Therefore, it seems that a combination of both nanoparticles and DESs contribute to the reaction progress. According to the results shown in Table 1, optimal conditions for the oxidation of benzylic alcohols to desired products were as follows: 50 mg of catalyst, solvent‐free, and H_2_O_2_ as oxidant at 60 °C.

**Table 1:** Optimization of the reaction conditions.

Entry	Cat. [mg]	Oxidant	Solvent	Temp. [°C]	Yield [%]
1	20	H_2_O_2_	Ethanol	RT	10
2	20	H_2_O_2_	–	60	50
3	50	H_2_O_2_	Ethanol	60	75
4	50	H_2_O_2_	–	60	90
5	50	H_2_O_2_	CH_3_CN	60	55
6	50	H_2_O_2_	Toluene	6 0	40
7	50	TBHP	–	60	70

Subsequently, substrate scope for the oxidation of various alcohols was investigated (Table 2).

**Table 2:** **Fe_3_O_4_@SiO_2_/**IPS catalyzed oxidation of various alcohols.^[a]^

Entry	Substrate	Product (Yield^[b]^ [%])	Time [h]
1	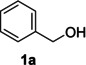	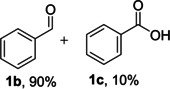	2
2	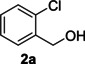	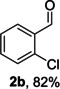	2
3	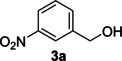	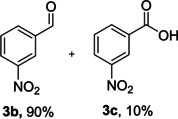	2
4	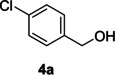	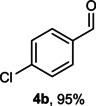	2
5	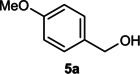	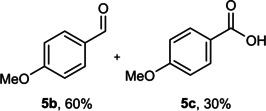	4
6	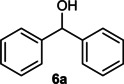	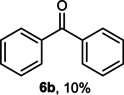	12
7	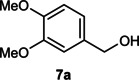	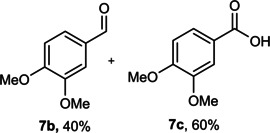	2
8	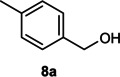	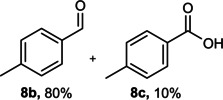	2
9	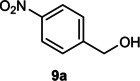	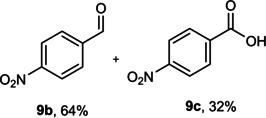	2
10	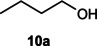	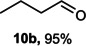	2
11	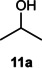	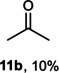	12
12	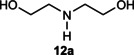	*	24
13	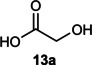	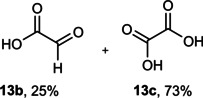	2
14	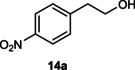	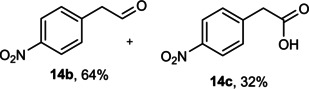	2

[a] Substrate (1 mmol), catalyst (50 mg), H_2_O_2_ (3 mmol) at 60 °C, under solvent‐free condition; [b] GC/MS yield based on the starting alcohol; * Impossible distinguishing of products.

The dominant product in all cases, except for 3,4‐dimethoxy benzyl alcohol (**7 a**) was the aldehyde. The highest yields of these benzylic hydroxyls (in the form of aldehydes) were attributed to (**3 a**) and (**4 a**). It can be referred to as kinetically‐active sites of benzyl alcohols with 3‐nitro and 4‐chloro substitutes which played a crucial role in having an electron‐enriched C−H bond for oxidation. However, just substrate (**3 a**) converted to the corresponding carboxylic acid (10 %) besides the main product. This observation could be explained as the tendency of this alcohol to pass through a thermodynamically favorable path to produce a further oxidation product. In the case of the presence of a chlorine atom in *ortho* (**2 a**) or *para* (**4 a**) positions, the hydroxyl group tended to be oxidized to just an aldehyde. In a conclusion, it can be comprehended that if there are one or more electron‐donating groups on the phenyl ring of benzyl alcohols, the dominant product will be carboxylic acids besides its aldehydes (**5 a** and **7 a**). By raising the number of electron‐donating groups on the phenyl ring of benzylic alcohol, the fraction of carboxylic acid to aldehyde becomes larger until it takes over one in OMe (**5 a**) with two methoxy substitutes (**7 a**). In 3,4‐dimethoxy benzyl alcohol (**7 a**), the main product was the corresponding carboxylic acid, which may be due to the presence of two strong electron‐donating substitutes in the *meta* and *para* positions. From a different viewpoint, either with or without a nitro group as an electron‐withdrawing substituent, the partial (10 %) oxidized carboxylic acid cannot be omitted. The secondary alcohol containing two aryl groups (**6 a**) afforded a 10 % yield under the optimized conditions.

Next, the catalytic oxidation of aliphatic alcohols was surveyed, and the highest conversion (95 %) was observed for butanol (**10 a**). Isopropyl alcohol (**11 a**) as secondary alcohol converted to the corresponding ketone in a longer reaction time with an overall 10 % yield. Diethanolamine (**12 a**) was not efficiently converted into the desired product. When 2‐hydroxyacetic acid (**13 a**) was treated under optimized conditions, oxalic acid was formed with a 73 % yield, accompanied by the formation of the corresponding aldehyde (25 %). Primary aliphatic alcohol (**14 a**) was oxidized more efficiently and the desired aldehyde was obtained in good yield (64 %) along with a moderate yield (32 %) of over oxidation product.

**Scheme 2:**
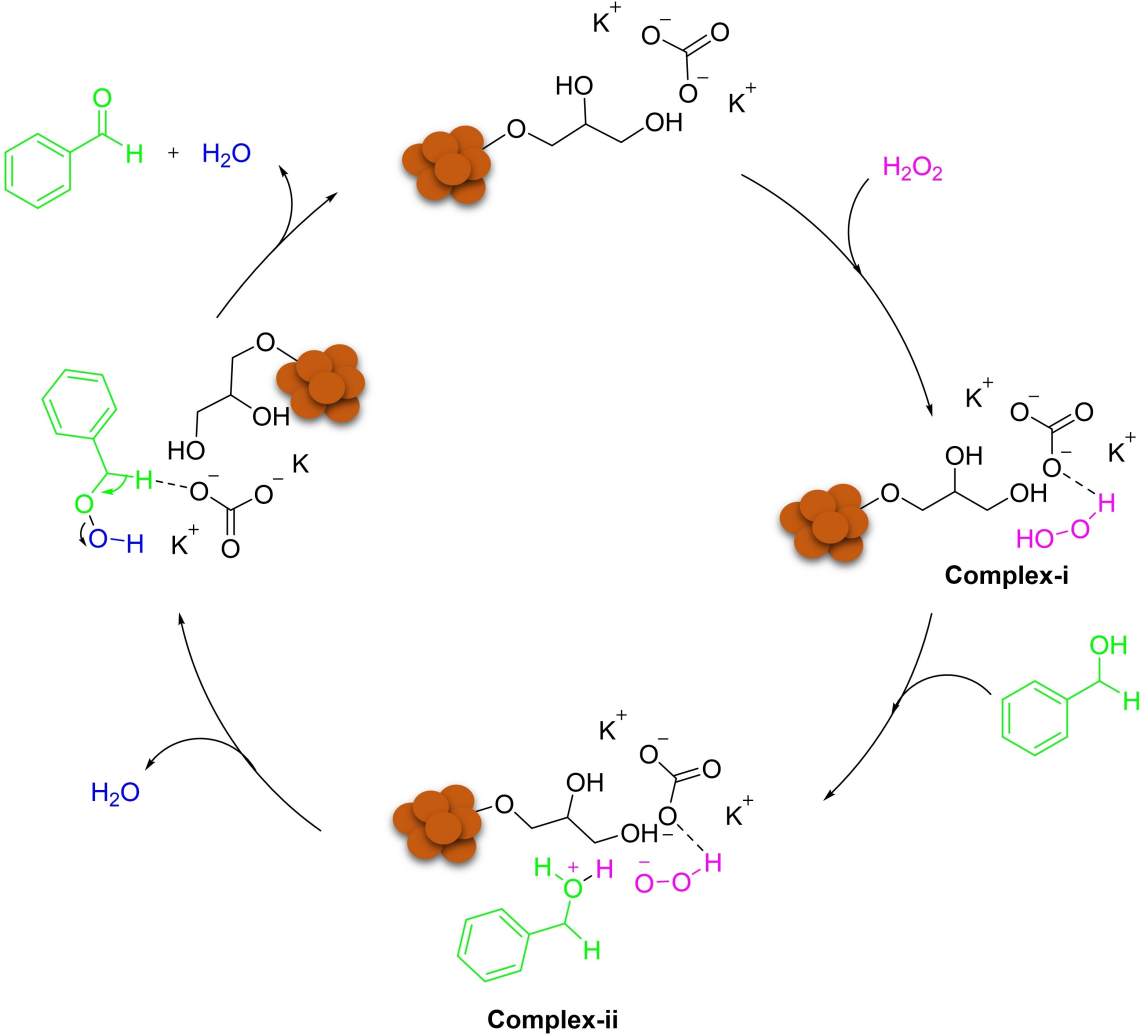
Possible reaction mechanism for the oxidation of benzyl alcohol.

According to the literature, the exact mechanism of the reaction is unclear.^[16]^ However, a possible mechanism was proposed as shown in Scheme 2. Initially, DES (K_2_CO_3_/Gly) forms a simple hydrogen bond with H_2_O_2_ providing complex‐I. Then, the activation occurs with the external proton of complex‐I to make complex‐II. Next, the OOH anion attacks the activated intermediate, and subsequently the benzyl hydroperoxide intermediate forms with the removal of H_2_O. Finally, the last intermediate undergoes deprotonation by basic media, and eventually, the product obtains with again removal of H_2_O.

The reusability of the prepared nanoparticles from the reaction medium was investigated. After the completion of the model reaction, the catalyst was separated by an external magnet and washed several times with water then dried at 80 °C for 24 h and used for the next run. The catalyst was reused three times without any significant loss of catalytic activity.

## Conclusions

In summary, we have developed a highly economical method for oxidation of alcohols to corresponding carbonyl products using the anchored K_2_CO_3_‐Gly to the modified magnetic nanoparticles as a green and efficient catalyst. The free‐metal catalytic oxidation of secondary and primary alcohols provides the corresponding carbonyl products and carboxylic acids, respectively, in good to high yields. Therefore, the unique design of Fe_3_O_4_@SiO_2_/IPS‐ K_2_CO_3_/Gly nanoparticles using earth‐abundant and inexpensive materials may offer an innovative approach for the exploration of highly active and stable heterogeneous catalysts for diverse catalytic applications.

## Experimental Section

### General

The solvents and starting materials were purchased from Merck (Germany) and Sigma‐Aldrich (USA). Fourier Transform Infrared (FT‐IR) spectra of the prepared samples were recorded on a Thermo AVATAR FT‐IR spectrometer in the wavenumber range of 400–4000 cm^−1^ using spectroscopic‐grade and pure KBr. Powder X‐ray diffraction (XRD) patterns were recorded at room temperature with a Philips X‐Pert 1710 diffractometer using Co Kα radiation (*λ*=1.78897 Å) at a voltage of 40 kV and current of 40 mA to study the crystalline structure of the nanocatalyst, data were recorded from 10° to 80° (2θ) with the scan speed of 0.05° s^−1^. The morphology of nanoparticles was studied using Scanning Electron Microscopy (SEM; TESCAN MIRA II) equipped with Energy‐Dispersive X‐ray (EDX) spectroscopy. Transmission Electron Microscopy (TEM) images were obtained using a CM120 apparatus with an accelerating voltage of 100 kV.

### Synthesis of 3‐iodopropyltrimethoxysilane (IPS)

The IPS was prepared according to the procedure reported by Tavakol.^[12a]^ For this, 3.5 mmol of 3‐chloropropyltrimethoxysilane (CPTMS) was mixed with NaI solution in acetone (15 mL, 0.233 m). The mixture was refluxed under argon flow for 24 h. To purify this activated organic linker, NaCl was removed by centrifugation, and the filtrate has been allowed to be dried. The yellow residue was dissolved in dichloromethane, filtered, and gradually evaporated to obtain the pure yellowish IPS.

### Immobilization of purified iodo‐activated organic linker on Fe_3_O_4_@SiO_2_

For this step, Fe_3_O_4_ nanoparticles were prepared through the co‐precipitation approach. Next silica shell formed on the outer layer of Fe_3_O_4_ MNPs using tetraethyl orthosilicate (TEOS) reagent in a basic aqueous medium with a pH of around 11.^[17]^ The as‐prepared Fe_3_O_4_@SiO_2_ nanoparticles are coated with IPS to obtain IPS‐functionalized Fe_3_O_4_@SiO_2_ (Fe_3_O_4_@SiO_2_/IPS) similar to Tavakol's method.^[12a]^ For this, the Fe_3_O_4_@SiO_2_ (1 g) was completely dispersed in 10 mL ethanol. In the next step, 4 mL IPS was added to the above mixture under stirring at 60 °C for about 8 h. The resulting precipitate was isolated from the reaction mixture by a magnet and cooled down. The final brown solid was washed several times with cold distilled water (4 °C) and acetone and dried to achieve the Fe_3_O_4_@SiO_2_/IPS.

### Synthesis of K_2_CO_3_‐Glycerol DES

The K_2_CO_3_‐Glycerol DES was prepared based on previous literature.^[10]^ K_2_CO_3_ and the Glycerol were mixed with a molar ratio of 1 : 10 in a round‐bottomed flask. The reaction temperature reached 80 °C under vigorous stirring for about 15 min.

### Synthesis of K_2_CO_3_‐Glycerol DES engrafted to Fe_3_O_4_@SiO_2_/IPS

Based on the reported Tavakol's group technique,^[12a]^ prepared Fe_3_O_4_@SiO_2_/IPS (0.1 g) and K_2_CO_3_‐Glycerol DES (2 g) were mixed in a 50 mL round‐bottomed flask, and the suspension was stirred for 18 h at 90 °C. The reaction mixture was then cooled down to room temperature. The resulting precipitate was separated using an external magnet, washed five times with distilled water, chloromethane, and acetone, and dried to afford the final catalyst (Fe_3_O_4_@SiO_2_/DES).

### General procedure for oxidation of alcohols

To the mixture of benzyl alcohol (1 mmol) and H_2_O_2_ (3 mmol) was added Fe_3_O_4_@SiO_2_/DES (50 mg) as the catalyst under free solvent and the resulting mixture was stirred at 60 °C until the reaction was complete, as monitored by TLC. After an appropriate time, the used nanocatalyst was separated from the reaction media and recovered. After an organic‐aqueous work‐up, the reaction mixture was injected into GC for an accurate analysis of the products.

## Conflict of interest

The authors declare no conflict of interest.

1

## Data Availability

The data that support the findings of this study are available from the corresponding author upon reasonable request.
